# Trajectory of Cardiogenic Dementia: A New Perspective

**DOI:** 10.1111/jcmm.70345

**Published:** 2025-01-19

**Authors:** Nawaf AlRawili, Hayder M. Al‐Kuraishy, Ali I. Al‐Gareeb, Maha M. Abdel‐Fattah, Nasser A. Al‐Harchan, Mubarak Alruwaili, Marios Papadakis, Athanasios Alexiou, Gaber El‐Saber Batiha

**Affiliations:** ^1^ Department of Internal Medicine, College of Medicine Northern Border University Arar Saudi Arabia; ^2^ Department of Clinical Pharmacology and Medicine, College of Medicine Mustansiriyah University Baghdad Iraq; ^3^ Department of Clinical Pharmacology Jabir ibn Hayyan Medical University Kufa Iraq; ^4^ Department of Pharmacology and Toxicology, Faculty of Pharmacy Beni‐Suef University Beni‐Suef Egypt; ^5^ Department of Clinical Pharmacology, College of Dentistry Al‐Rasheed University Baghdad Iraq; ^6^ Department of Internal Medicine, College of Medicine Jouf University Saudi Arabia; ^7^ Department of Surgery II University Hospital Witten‐Herdecke, University of Witten‐ Herdecke Wuppertal Germany; ^8^ University Centre for Research & Development Chandigarh University Mohali India; ^9^ Department of Science and Engineering Novel Global Community Educational Foundation New South Wales Australia; ^10^ Department of Research & Development Athens Greece; ^11^ Department of Research & Development AFNP Med Wien Austria; ^12^ Department of Pharmacology and Therapeutics, Faculty of Veterinary Medicine Damanhour University Damanhour AlBeheira Egypt

**Keywords:** cardiogenic dementia, cognitive impairment, heart failure, myocardial infarction and atrial fibrillation

## Abstract

The functions of the heart and brain are closely linked and essential to support human life by the heart‐brain axis, which is a complex interconnection between the heart and brain. Also, cardiac function and cerebral blood flow regulate the brain's metabolism and function. Therefore, deterioration of cardiac function may affect cognitive function and may increase the risk of dementia. Cardiogenic dementia is defined as a cognitive deterioration due to heart diseases such as heart failure, myocardial infarction, and atrial fibrillation. The prevalence of cognitive impairment in patients with heart failure was 29%. In addition, coronary artery disease (CAD) is also associated with the development of cognitive impairment. CAD and reduction of myocardial contractility reduced cerebral blood flow and increased the risk of dementia in CAD patients. Furthermore, myocardial infarction and subsequent systemic haemodynamic instability promote the development and progression of cardiogenic dementia. These findings indicated that many cardiac diseases are implicated in the development and progression of cognitive impairment. Nevertheless, the underlying mechanism for the development of cardiogenic dementia was not fully elucidated. Consequently, this review aims to discuss the potential mechanisms involved in the pathogenesis of cardiogenic dementia.

## 1 Introduction

Cardiogenic dementia is defined as a cognitive deterioration due to heart diseases such as heart failure, myocardial infarction, and atrial fibrillation [1]. The term cardiogenic dementia was first described by Lancet in 1977 as cognitive impairment caused by different cardiac diseases [1]. Cognitive impairment is common among patients with acute or chronic heart failure due to reduction of cerebral blood flow [2]. It has been observed that acute heart failure may cause mild to severe cognitive impairment [3]. Increasing of cardiovascular risk predicts deterioration in memory and speed of working memory due to the development of vascular lesions and associated brain neurodegeneration [4]. In turn, cognitive impairment may increase the risk of cardiovascular complications [5]. The severity of cognitive impairment is regarded as an independent risk factor for high mortality in patients with acute heart failure [6]. The prevalence of cognitive impairment in patients with heart failure is 20%–80% [7]. This wide‐range fluctuation may be due to various factors, such as screening tests, sample size, and diversity of study design that affect the prevalence of cognitive impairment in patients with heart failure [7]. In the early 2000s, the prevalence of cognitive impairment in patients with heart failure was around 35% [8]. In 2007, the prevalence of mild cognitive impairment in patients with heart failure was 61%, though the prevalence of severe cognitive impairment was 31% [9]. However, a recent study revealed that the prevalence of cognitive impairment in patients with heart failure was 29% [10].

In addition, coronary artery disease (CAD) is also associated with the development of cognitive impairment [11] CAD and reduction of myocardial contractility reduce cerebral blood flow and increase the risk of dementia in CAD patients compared to healthy controls [12]. It has been shown that CAD patients had low serum levels of brain‐derived neurotrophic factor (BDNF), which has a cognitive enhancer effect and negatively correlates with the risk of cognitive impairment and Alzheimer's disease (AD) [13]. Furthermore, atrial fibrillation, by increasing the risk of recurrent ischaemic stroke predisposes for the development of cognitive impairment and dementia [14]. Ischaemic stroke augments risk for the development of vascular dementia and AD by increasing the production of amyloid beta (Aβ) in response to the ischemic ents [15]. Importantly, activation of platelets in atrial fibrillation is correlated with cognitive impairment and the development of AD either directly by releasing amyloid precursor protein (APP) or indirectly by the release of pro‐inflammatory cytokines [16]. Therefore, anticoagulant and antiplatelet agents attenuate cognitive impairment in patients with atrial fibrillation [17]. Interestingly, atrial fibrillation triggers inconsistency in cerebral haemodynamic with subsequent reduction of cerebral blood flow [18].

Moreover, myocardial infarction and subsequent systemic haemodynamic instability promote the development and progression of cardiogenic dementia. A multicenter study observed that 29.8% of patients with myocardial infarction develop cognitive impairment [19, 20]. Peripheral vasoconstriction and hypoperfusion in patients with myocardial infarction reduced cerebral blood flow and the development of brain ischaemic changes [21, 22]. Notably, myocardial infarction increases risk for the development of vascular dementia rather than AD due to alteration of cerebral vasculature [21, 23].

The functions of the heart and brain are closely linked and essential to support human life. The heart‐brain axis is a complex interconnection between the heart and brain; sympathetic and parasympathetic outflow from brain cortical and subcortical regions affects cardiovascular function. Furthermore, the metabolism and function of the brain are regulated by heart activity and cerebral blood flow [24, 25]. The presence of cardiac fibrosis is associated with the progression of myocardial infarction and death. Cardiac fibrosis is prevalent in almost all types of heart illness, in particular in individuals who experience a myocardial infarction and those with both ischaemic and non‐ischaemic cardiomyopathies [26, 27]. In these cases, fibrosis is linked to the depletion of viable myocardium.

Therefore, brain diseases such as neurodegenerative diseases and cerebrovascular diseases may cause myocardial infarction and cardiac dysfunction [28]. A systematic review and meta‐analysis have shown that stroke increases the risk of myocardial infarction [29]. However, cognitive function and memory tasks are affected by cardiac function [24, 25]. Moreover, biomarkers of myocardial injury are correlated with brain injury and cognitive impairment in elderly patients [23, 30].

**FIGURE 1:**
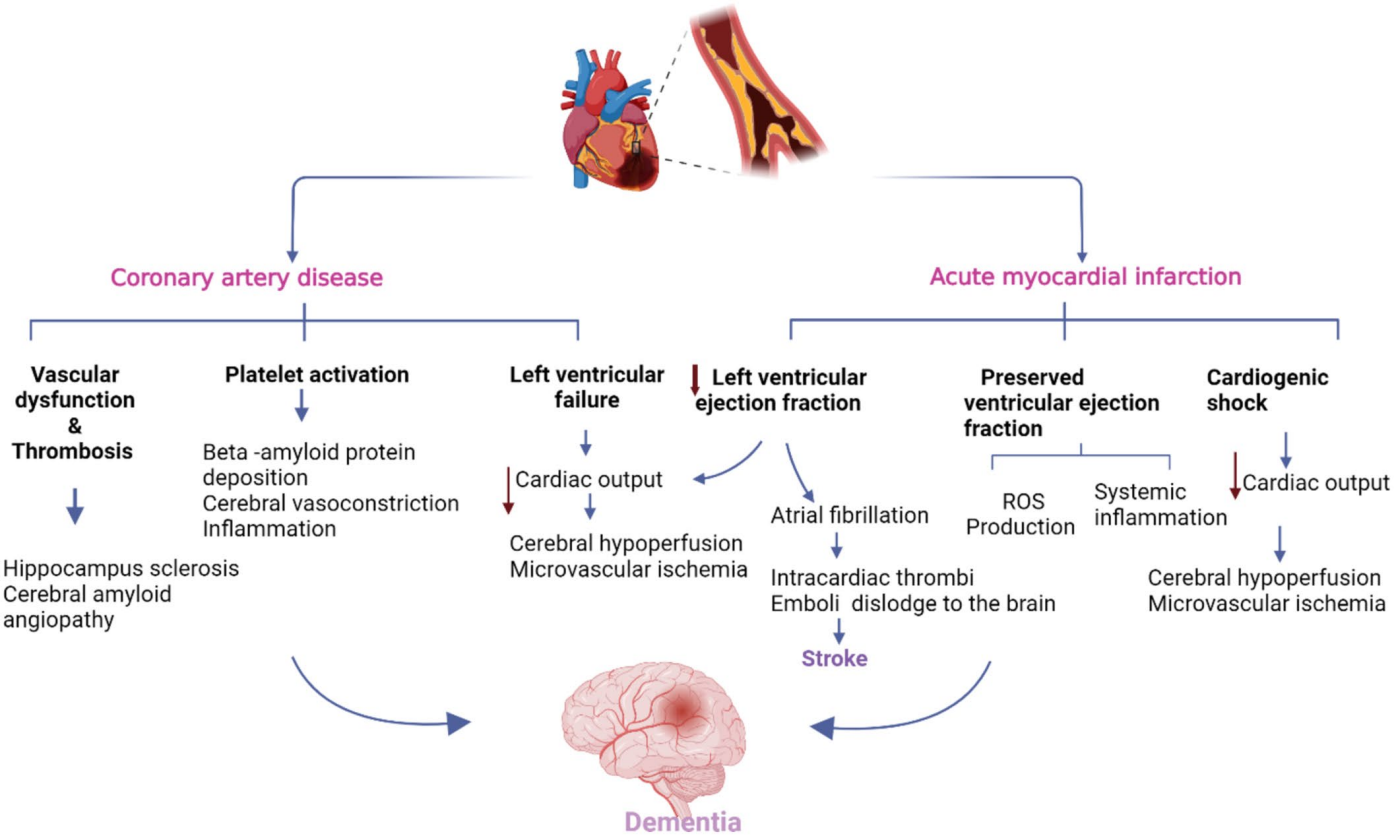
Pathways of cardiogenic dementia (Created with BioRender.co).

These findings indicated that many cardiac diseases are implicated in the development and progression of cognitive impairment (Figure 1). However, the underlying mechanism for the development of cardiogenic dementia was not fully elucidated. Therefore, this review aims to discuss the potential mechanisms involved in the pathogenesis of cardiogenic dementia.

## 2 Pathophysiology of Cardiogenic Dementia

Different mechanisms are involved in the pathogenesis of cardiogenic dementia, such as chronic cerebral hypoperfusion‐dependent mechanisms and chronic cerebral hypoperfusion‐independent mechanisms.

### 2.1 Chronic Cerebral Hypoperfusion

Chronic cerebral hypoperfusion is caused by heart failure, atherosclerosis, carotid stenosis, and chronic hypotension [31]. Chronic cerebral hypoperfusion induces the development and progression of neurodegeneration, neurocognitive disorders, and cognitive decline [31]. It has been reported that chronic cerebral hypoperfusion triggers the development of cognitive impairment before the development of AD neuropathology [32]. In addition, cardiovascular risk factors such as cardiometabolic disorders and low cardiac index reduce cerebral hypoperfusion and promote the development of AD and progression of cardiogenic dementia [32]. Findings from preclinical studies revealed that chronic cerebral hypoperfusion augments AD neuropathology and the development of cognitive impairment by increasing the production of Aβ and formation of amyloid aggregates, which further disturb cerebral microcirculation and derange the BBB [33, 34]. Findings from postmortem human brains and experimental cerebral amyloid angiopathy (CAA) mouse models demonstrated that chronic cerebral hypoperfusion was correlated with microinfarcts and the development of CAA and Aβ aggregates. In the experimental bilateral carotid artery stenosis, Aβ aggregates were increased in APP transgenic mice compared to controls [34]. Moreover, brain ischaemia and hypoxia due to chronic cerebral hypoperfusion augments the activity of β‐secretase (BACE1) through induction of the expression of the *BACE1* gene by hypoxia‐inducible factor‐1α in transgenic mice [35]. Overactivation of BACE1 increased the production of Aβ through the amyloidogenic pathway in mice subjected to brain energy insufficiency [36]. Furthermore, brain ischaemia augments the susceptibility of neurones to the neurotoxic effect of Aβ through dysregulation of calcium homeostasis, which is also dysregulated in AD and other dementia types [37]. As well, chronic cerebral hypoperfusion after ischaemic stroke interferes with clearance of Aβ via the glymphatic pathway, leading to the development of post‐stroke dementia in a rat model [38]. These findings indicated that chronic cerebral hypoperfusion promotes AD neuropathology by activating the amyloidogenic pathway and inhibiting Aβ clearance.

Furthermore, chronic cerebral hypoperfusion promotes the development of cerebral small vessel disease leading to brain ischaemia, lacunar infarction, white matter lesions, and progression of vascular dementia [39]. In addition, chronic cerebral hypoperfusion caused by congestive heart failure, atrial fibrillation, and CAD triggers the development of AD [40]. A longitudinal, population‐based study, the CAIDE study, followed up patients with cardiac diseases for 25 years and showed that 8.4% developed dementia. In addition, both late‐life heart failure and atrial fibrillation, but not CAD, were the potential independent factors involved in the development of dementia, mainly in ApoE4 carriers [40]. Therefore, late‐life heart failure and atrial fibrillation should be treated to prevent the development of dementia. Of note, chronic cerebral hypoperfusion induces retention of CO_2_ leading to cerebral vasodilation, though this compensatory mechanism is limited in elderly patients with heart failure [41]. The vascular reactivity is impaired in patients with heart failure, leading to the reduction of cerebral blood flow [42]. Moreover, overactivation of the renin‐angiotensin‐aldosterone system (RAAS) and vasopressin in heart failure can cause cerebral vasoconstriction leading to chronic cerebral hypoperfusion [43, 44].

Interestingly, chronic cerebral hypoperfusion leads to brain glucose hypometabolism before the development of dementia. Brain glucose hypometabolism results in neuronal energy dysfunction, BBB injury, endothelial dysfunction, and Aβ formation [45]. Chronic cerebral hypoperfusion and brain glucose hypometabolism trigger neurodegeneration, synaptic dysfunction, neuronal apoptosis, and brain atrophy [45]. Brain glucose hypometabolism is common in patients with cognitive impairment and AD and is involved in the progression of dementia neuropathology [46]. Additionally, brain glucose hypometabolism is also reduced in patients with frontotemporal dementia [47]. Brain glucose hypometabolism can cause neurotoxicity and hyperexcitability by inducing calcium dyshomeostasis and glutamate release, which further induce neurodegeneration [38].

**FIGURE 2:**
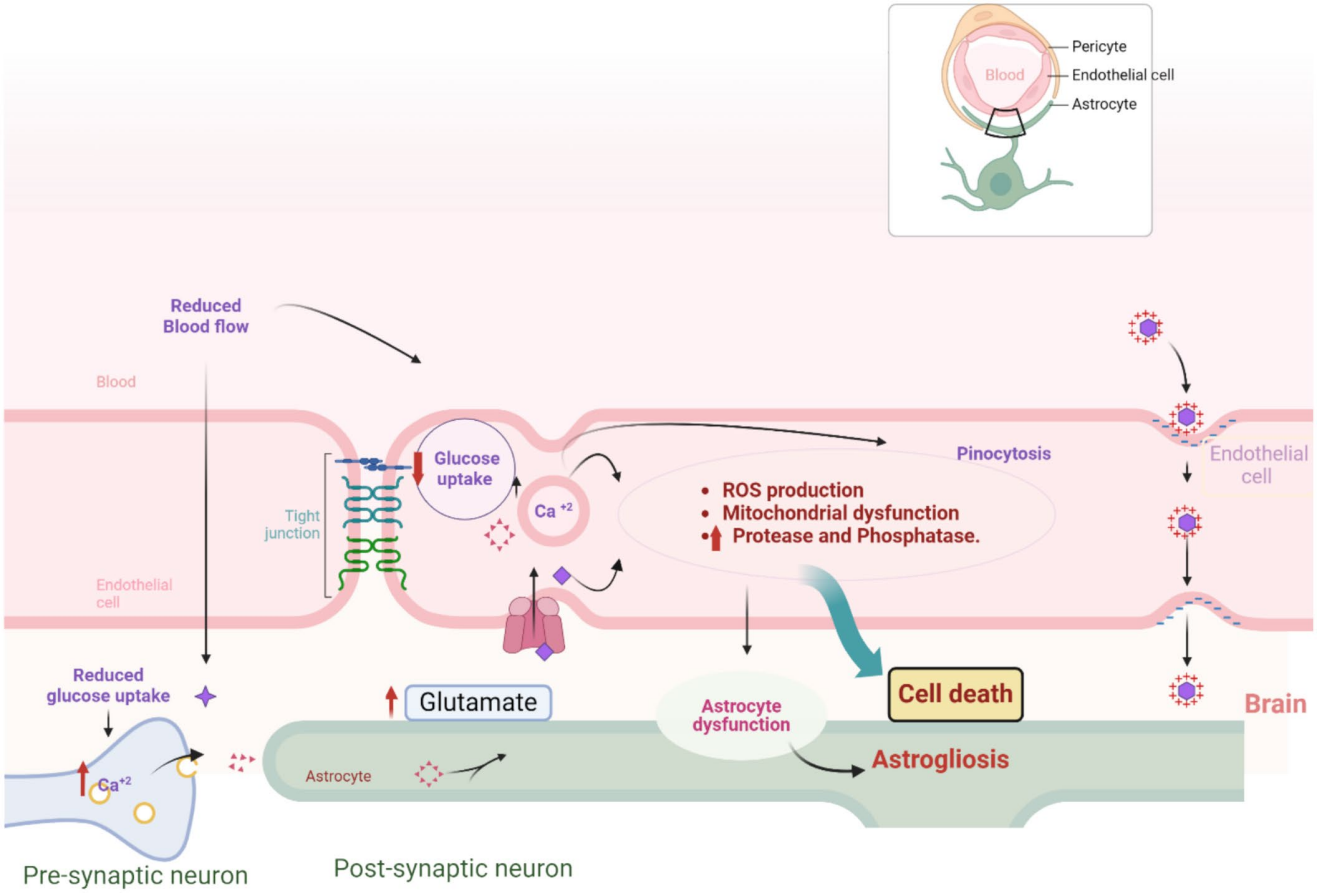
Chronic cerebral hypoperfusion and the development of dementia: As reduced cerebral blood flow is associated with a number of mechanisms that affect endothelial cells and adjacent cells. One such mechanism is excitotoxicity, which is induced in endothelial cells in response to a decrease in glucose intake. Abnormally high levels of cytosolic Ca^2+^ are linked to metabolic disruption, mitochondrial dysfunction, activation of proteases and phospholipases, and production of reactive oxidative species (ROS) that all work together to cause cell membrane damage and vascular cell death, which ultimately compromises the integrity of the blood–brain barrier. Excitotoxins negatively impact BBB astrocytes by quickly disrupting the connection between astrocytes and the extracellular matrix (ECM). Mostly, after excitotoxicity, astrogliosis or malfunctioning of the astrocytes combined with increased transendothelial pinocytosis causes the BBB to break down (Created with BioRender.co).

These findings indicated that chronic cerebral hypoperfusion due to different cardiac diseases induces the development of dementia by inducing Aβ formation, neurodegeneration, and brain glucose hypometabolism (Figure 2).

### 2.2 Chronic Low‐Grade Inflammation

Chronic low‐grade inflammation is common in heart failure due to sustained activation of the nod‐like receptor pyrin 3 (NLRP3) inflammasome, which mediates the progression of heart failure and cardiovascular complications in the elderly [48]. In addition, heart failure with reduced ejection fraction is linked with high levels of circulating pro‐inflammatory cytokines [49]. Heart failure induces the development of chronic low‐grade inflammation, and targeting of inflammation by anti‐cytokine monoclonal antibody mitigates the clinical outcomes in patients with heart failure consequent to myocardial infarction [49, 50].

Moreover, chronic low‐grade inflammation is interrelated with coronary artery disease and atrial fibrillation [51, 52]. Patients with atrial fibrillation are associated with elevated inflammatory biomarkers [52]. Furthermore, chronic low‐grade inflammation is implicated in the pathogenesis of dementia by inducing taupathy and accumulation of Aβ [53]. In addition, chronic inflammation enhances the hyperphosphorylation of tau protein and the formation of neurofibrillary tangles (NFTs), a hallmark of AD [53]. Interestingly, chronic low‐grade inflammation in obesity and metabolic syndrome can induce the development of AD by injury of the BBB, impairment of synaptic plasticity, induction of synaptic injury, and neuronal apoptosis [54, 55]. Importantly, plasma levels of inflammatory biomarkers are correlated with functional disability in patients with AD or mixed dementia compared to healthy controls [56, 57]. A cohort study indicated that high levels of systemic inflammatory biomarkers augment and hasten the development of AD. However, a high level of systemic low‐grade inflammation biomarkers is not associated with cognitive impairment in middle aged patients [58]. Therefore, the effect of systemic low‐grade inflammation may be more in the old‐age group in the induction of cognitive impairment. A systematic review and meta‐analysis involving 14 studies highlighted that a high level of the inflammatory biomarker CRP was not correlated with risk of cognitive impairment but predicted the conversion to dementia [59]. Indeed, systemic low‐grade inflammation induces the development of neuroinflammation through activation of microglia with subsequent development of cognitive dysfunction [60].

**FIGURE 3:**
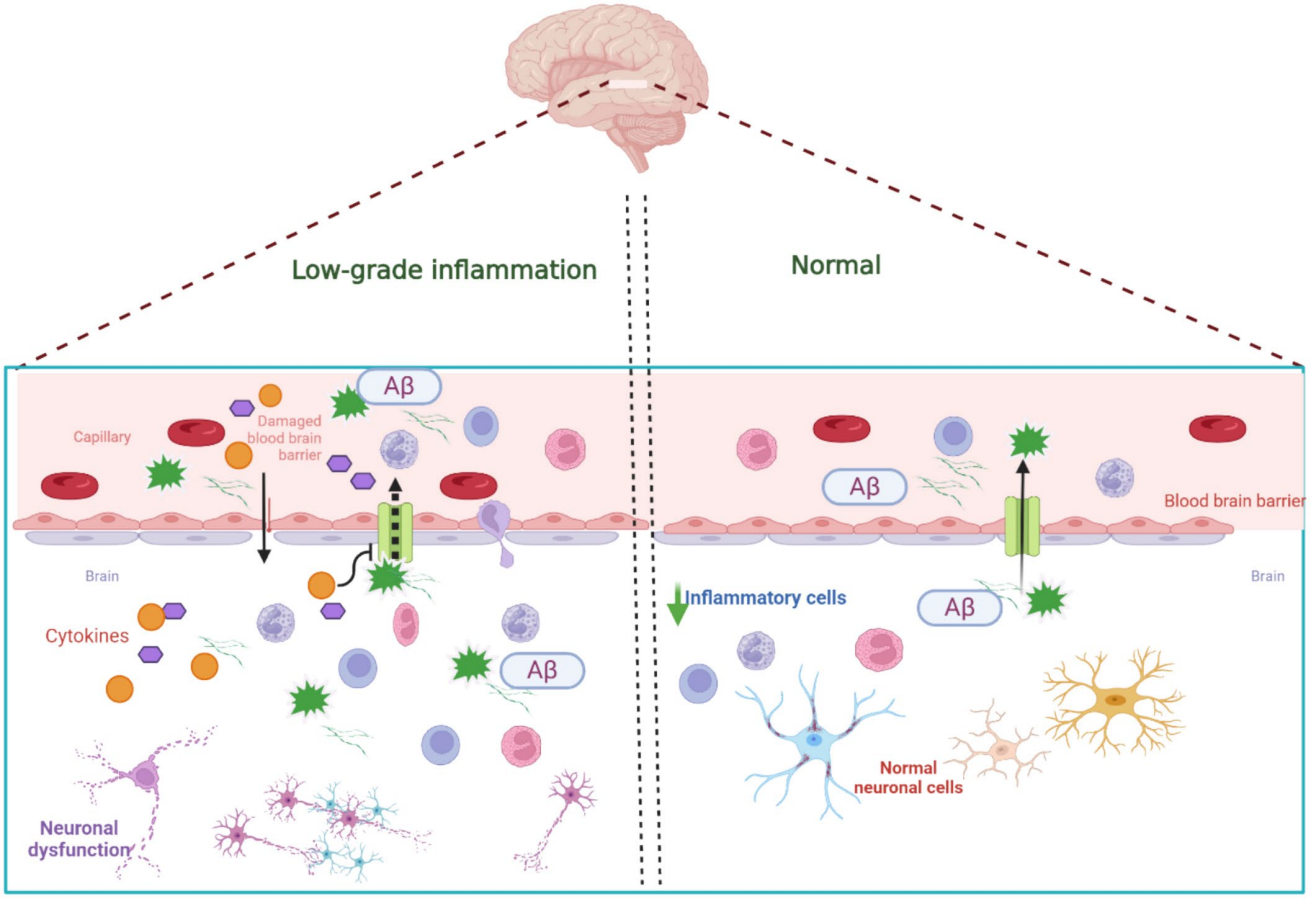
Low‐grade peripheral inflammation role in induction of dementia (Created with BioRender.co).

These verdicts pointed out that systemic low‐grade inflammation in cardiac diseases may be implicated in the pathogenesis of cardiogenic dementia (Figure 3).

The right side of the figure showed the normal state (with blood–brain barrier integrity), where amyloid β (Aβ) protein was not accumulated inside the brain. However, on the left side (inflammation), the inflammatory cytokines prevent the efflux of Aβ, causing insufficient Aβ clearance, promotion of Aβ aggregation, and neuronal dysfunction.

### 2.3 Oxidative Stress

Oxidative stress is a state of redox imbalance characterised by overproduction of ROS and depletion of endogenous antioxidant capacity [61, 62, 63]. Many studies illustrated that oxidative stress and mitochondrial dysfunction are involved in the development and progression of heart failure, atrial fibrillation, and CAD [52, 64, 65]. It has been stated that biomarkers of oxidative stress, such as NADPH oxidase, myeloperoxidase, and advanced glycation end‐products (AGEs), are augmented in patients with heart failure [66]. The plasma levels of oxidative stress biomarkers are correlated with the severity of heart failure in patients with dilated cardiomyopathy [67]. In heart failure, the production of ROS is augmented while the antioxidant defence mechanism is reduced in myocardium [68]. In patients with heart failure, both inflammation and endothelial dysfunction promote the generation of ROS and the development of oxidative stress [69].

**FIGURE 4:**
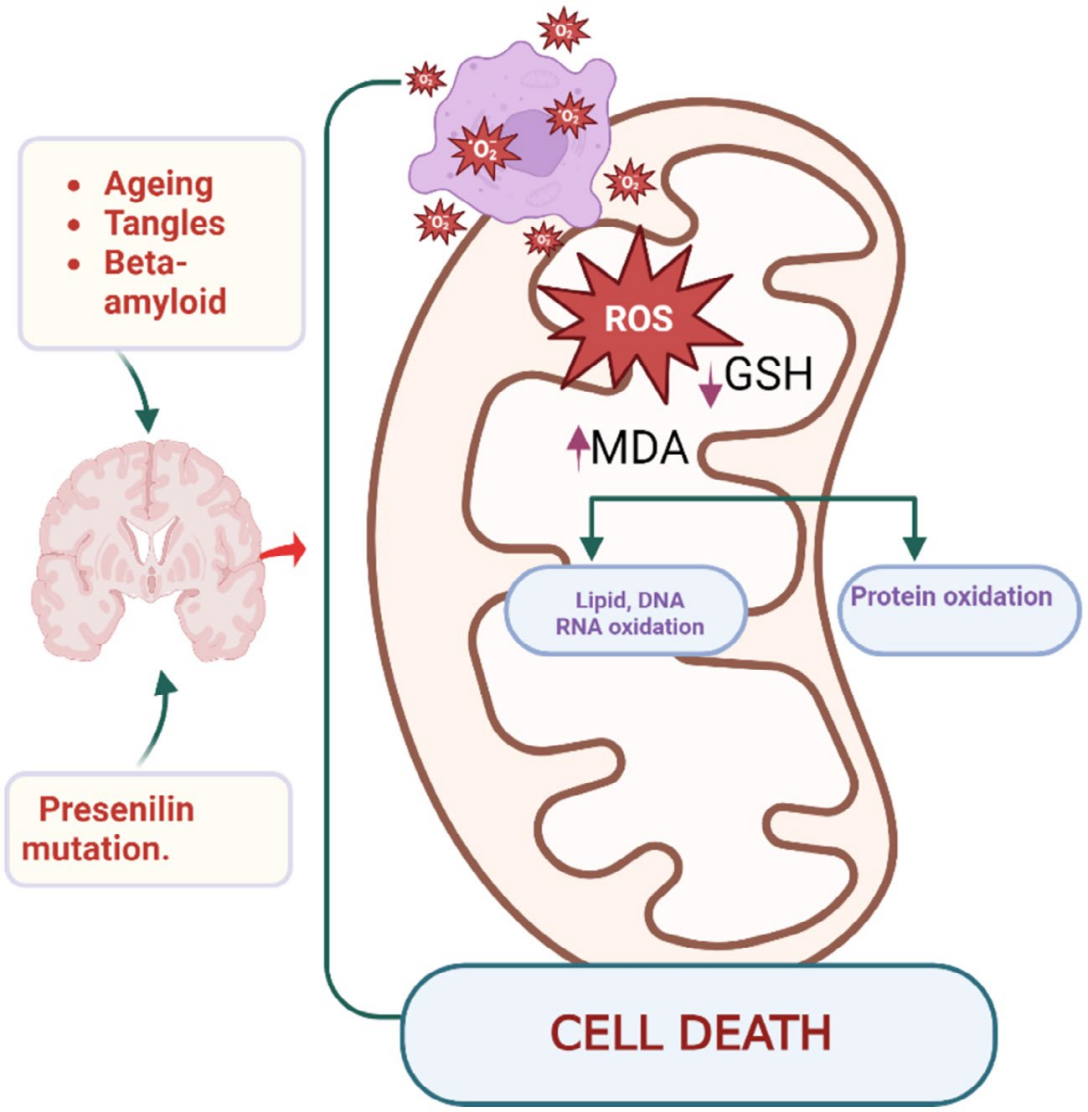
Oxidative stress role in the progression of dementia (Created with BioRender.co).

Furthermore, systemic oxidative stress is implicated in the conversion of mild cognitive impairment to late‐onset AD [70]. The biomarker of lipid peroxidation, such as MDA, was augmented, whereas plasma levels of antioxidant capacity were reduced in patients with mild cognitive impairment to late‐onset AD compared to healthy controls [70]. Indeed, systemic oxidative stress develops before the onset of AD neuropathology [71] suggesting its involvement in the pathogenesis of AD and other types of dementia. Therefore, the use of antioxidants can mitigate the progression of dementia [71]. Oxidative stress may also induce systemic inflammation and, more specifically neuroinflammation, which may contribute to the development of dementia [72]. Oxidative stress promotes the production and aggregation of Aβ and NFTs, which further induce its progression by inhibiting the antioxidant enzymes [72, 73]. Moreover, oxidative stress causes injury to DNA and cellular structures directly or through activation of microglia and development of neuroinflammation [74]. Aβ acts as a damage‐associated molecular pattern (DAMP) that induces the activation of microglia, which are the major source of ROS in the brain [75]. Therefore, augmentation of systemic oxidative stress in different cardiac diseases may be related to the development and progression of cardiogenic dementia (Figure 4).

### 2.4 Blood–Brain Barrier Injury

The BBB plays a critical role in the regulation of the brain's metabolic function and preserves the homeostasis of the microenvironment [76]. The systemic inflammation induced by congenital heart disease combined with oxidative stress and hypoxia causes BBB injury to the newborn [77]. A prospective study revealed that CSF albumin increased by 13%, serum albumin decreased by 27%, and the CSF/serum albumin ratio increased by 61% in patients undergoing cardiac surgery [78]. Therefore, cardiopulmonary bypass and associated hypoxaemia during cardiac surgery cause BBB injury and neuronal damage. Moreover, myocardial infarction and heart failure may cause BBB injury by different mechanisms, including hypoxia, cerebral hypoperfusion, development of systemic oxidative stress and inflammation, glial activation, neurohormonal activation, and the development of neuroinflammation [24]. The BBB injury facilitates the entry of immunoinflammatory cells into the brain leading to neuroinflammation, brain oxidative stress, and the induction the development of dementia [79]. Moreover, BBB injury exacerbates coronary atherosclerosis and ischaemic‐reperfusion injury by enhancing the transport of Aβ from the brain to the systemic circulation [80]. Remarkably, Aβ1‐42 can cause coronary endothelial injury and cytotoxicity of cardiomyocytes [81]. Therefore, there is a close relationship between the brain and heart via the heart brain axis.

**FIGURE 5:**
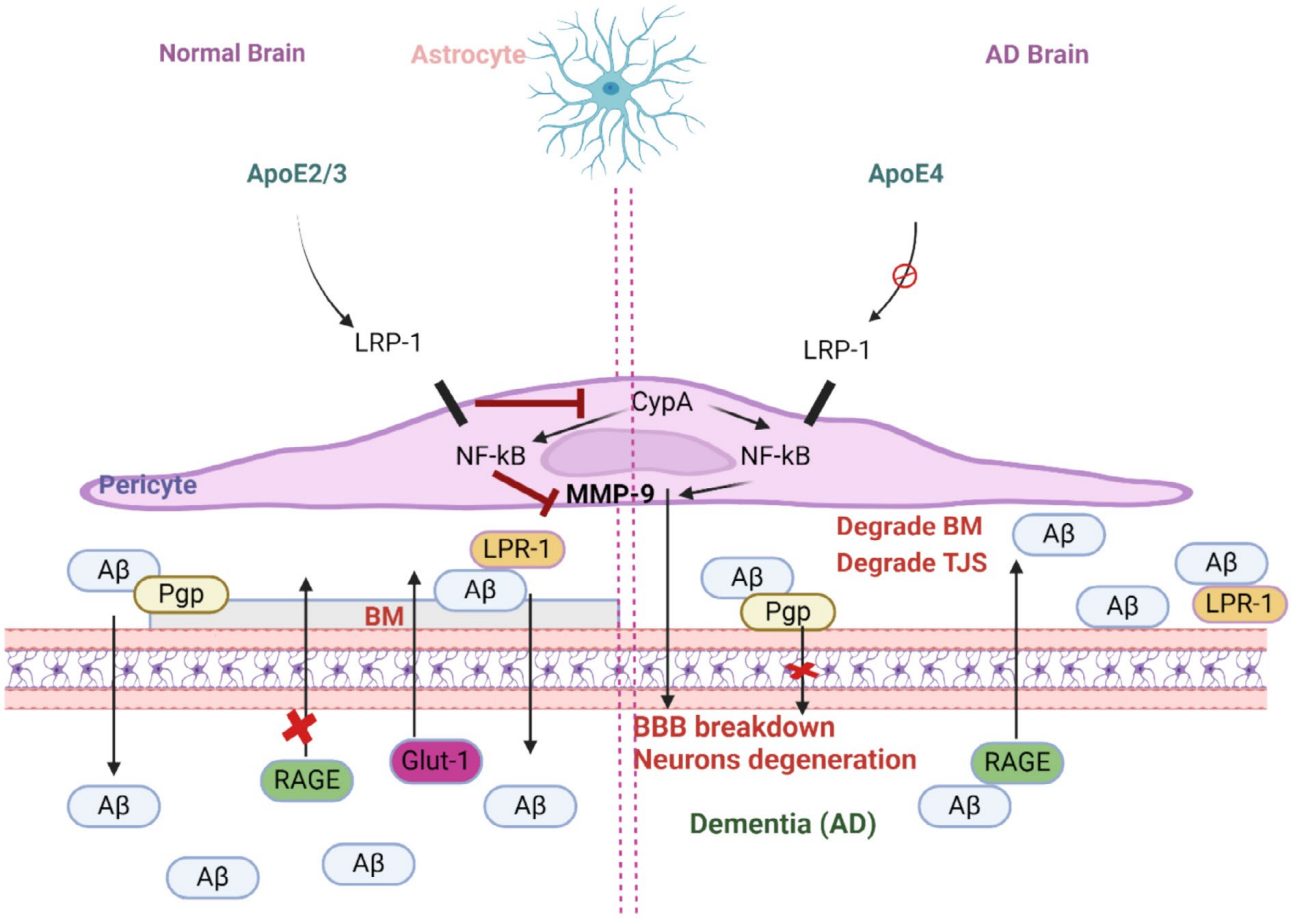
The blood–brain barrier (BBB) breakdown processes in a normal brain and an Alzheimer's disease (AD) brain are depicted in a schematic diagram (created with BioRender.co).

Furthermore, impairment of BBB integrity is associated with the development of dementia by increasing peri‐vascular inflammation with progressive accumulation of misfolded proteins [79]. During ageing, the BBB is impaired, and there is an increased risk for the development of cognitive impairment, AD and other dementias [82]. Therefore, BBB injury is regarded as an initial step in the development of dementia [83]. Reduction in the clearance of Aβ from the brain causes diminution of cerebral blood flow leading to BBB ischaemia and disruption [84, 85]. As well, Aβ‐induced oxidative stress and inflammation further disturb the integrity of the BBB in the AD model [86] (Figure 5).

A normal brain: In order to maintain the integrity of BBB and the BM, astrocytes release ApoE2/3, bind to low‐density lipoprotein receptor‐related protein‐1 (LRP‐1) on pericytes, and repress CypA‐NF‐kB. This, in turn, stops the secretion of matrix metalloproteinase 9 (MMP‐9) in pericytes. Additionally, endothelial cells' LRP‐1 and Pgp aid in the clearance of Aβ. To prevent the transfer of Aβ into the brain, the expression of the receptor for advanced glycosylation end products (RAGE) is suppressed. In the AD brain, astrocytes secrete ApoE4, and they also weakly interact with pericytes' LRP‐1, activating CypA‐NFkB‐MMP‐9 pathways. This leads to the degradation of BM and tight junctions (TJs), which breaks down the BBB and is linked to dementia and neurodegeneration. Additionally, ApoE4 interacts weakly with LRP‐1 on ECs, which is unable to effectively remove Aβ from the brain. As a result, Aβ builds up in the brain and damages neurons. Moreover, there is an upregulation of RAGE expression, which facilitates the transfer of Aβ from blood to the brain. Neurotoxins and blood cells permeate the brain, leading to dementia and neuronal degeneration.

**FIGURE 6:**
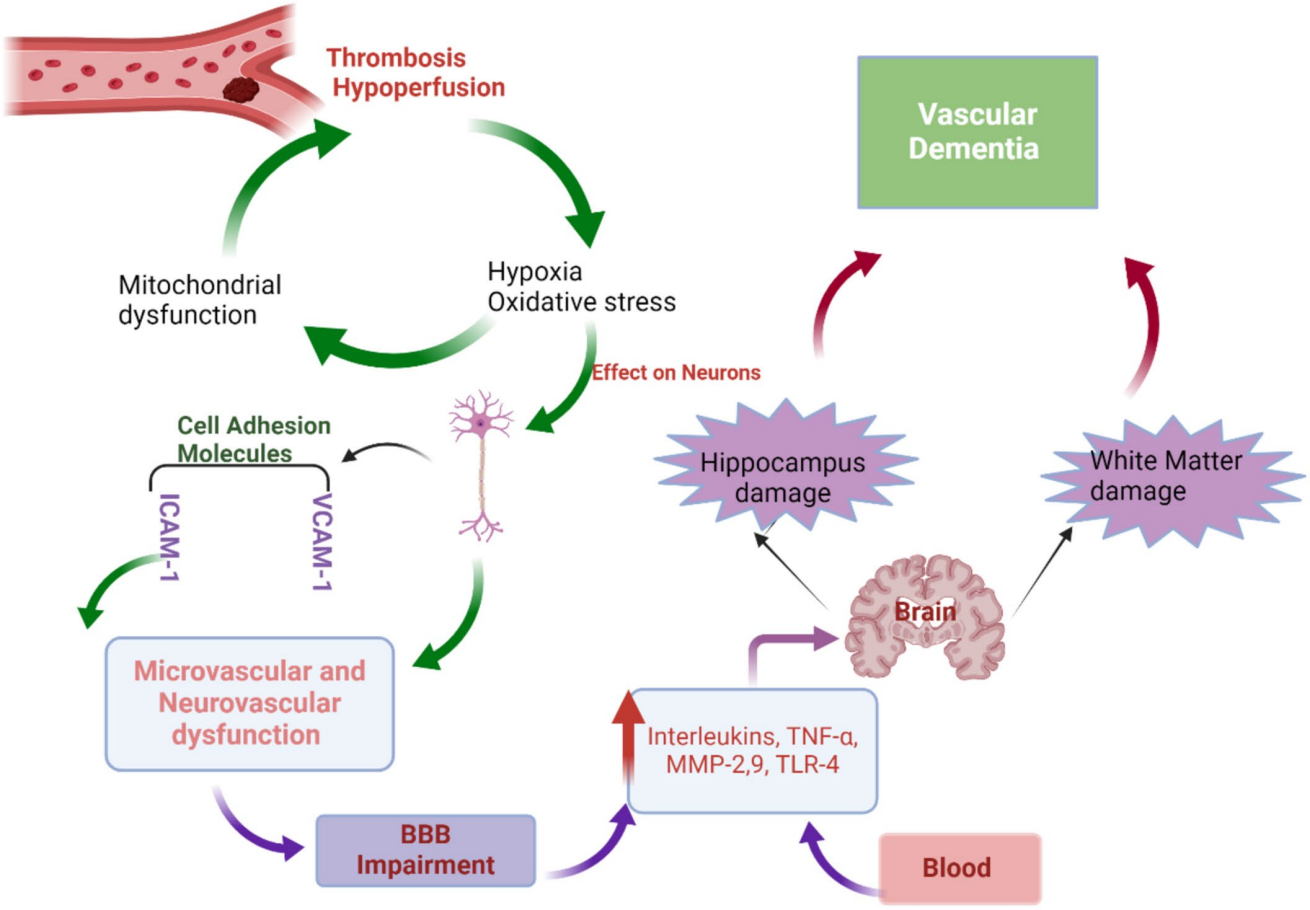
BBB injury in vascular dementia through induction of oxidative stress and inflammation (Created with BioRender.co).

However, thrombosis and hypoperfusion contribute to BBB injury in vascular dementia through the induction of oxidative stress and inflammation [87] (Figure 6).

Therefore, BBB injury induced by cardiac diseases could be a more potential mechanism in the development and progression of dementia.

### 2.5 Overactivation of Renin Angiotensin Aldosterone System

**FIGURE 7:**
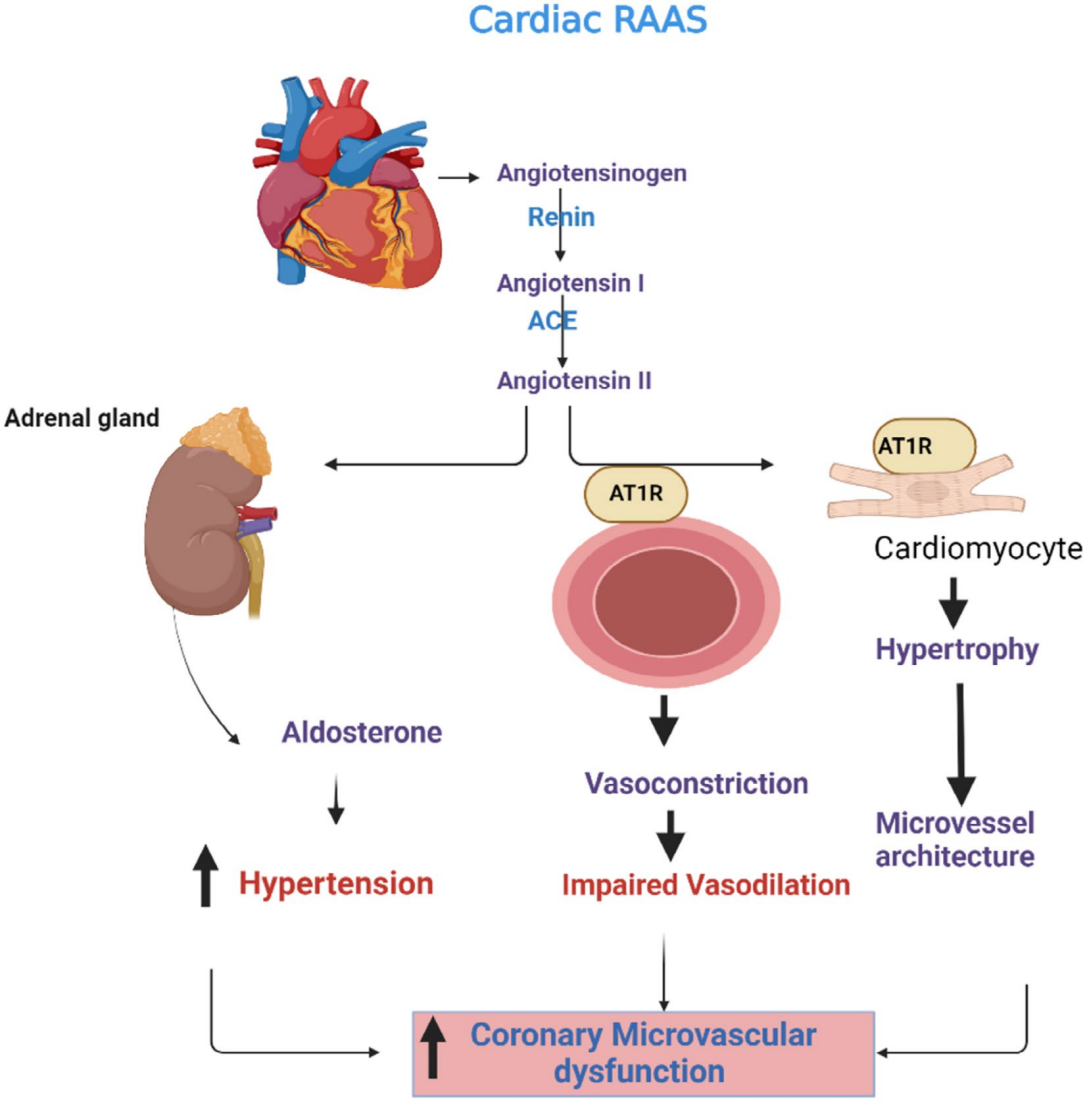
Role of Cardiac RAAS in Dementia (Created with BioRender.co).

Renin‐angiotensin‐aldosterone system (RAAS) is a neuroendocrine system involved in the regulation of blood pressure, salt and water content, cardiac contractility, and cognitive function [88]. Reduction of cardiac output, as in heart failure, triggers the activation of RAAS. Angiotensin protein released from the liver is converted to angiotensin I (AngI) by renin produced by the kidney. The angiotensin‐converting enzyme (ACE) converts AngI to AngII [89]. In turn, AngII, via activation of angiotensin receptor type 1 (AT1R), leads to vasoconstriction, cardiomyocyte activation, and stimulation of the release of aldosterone from the adrenal cortex [90, 91, 92]. In a similar manner, cardiac RAAS also produces local and systemic effects [93]. Cardiac RAAS is overactivated in hypertension and heart failure, leading to local and systemic inflammatory deleterious effects [93]. Activation of RAAS is regarded as a compensatory mechanism to preserve cardiac function and blood pressure in heart failure. The inflammatory role of RAAS is counteracted by the anti‐inflammatory axis, such as alamandine, Ang1‐7, and Ang1‐9 [89]. In addition, RAAS is involved in the pathogenesis of acute coronary syndrome and myocardial infarction [94, 95]. Therefore, exaggerated response of RAAS in many cardiac diseases may induce systemic inflammatory and oxidative stress reaction [96] (Figure 7).

**FIGURE 8:**
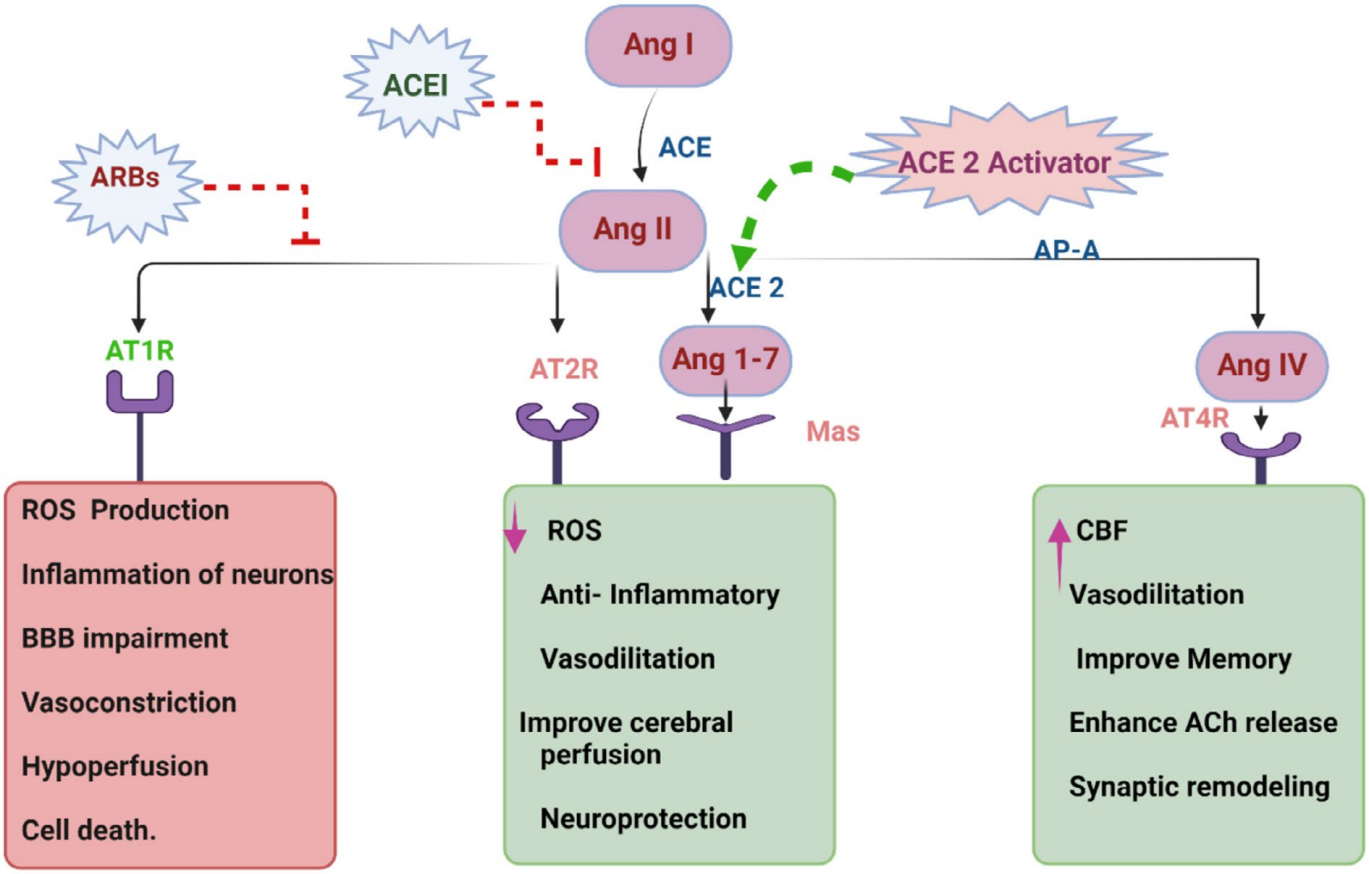
Targeting of RAAS in dementia (Created with BioRender.co).

Furthermore, overactivated RAAS in cardiac diseases may induce the development and progression of cognitive impairment and dementia [97]. Activation of AT1R is associated with BBB injury and neuroinflammation in mice [98]. Furthermore, brain AngII contributes to the pathogenesis of vascular dementia by inducing oxidative stress, neuroinflammation, synaptic injury, and endothelial dysfunction [99]. Both central and peripheral RAAS are associated with the progression of AD neuropathology by increasing the production and impeding the clearance of Aβ [44]. Thus, ACE inhibitors and angiotensin receptor blockers (ARBs) may reduce the pathogenesis of dementia and cognitive impairment by reducing neuronal inflammation and oxidative stress [100] (Figure 8). A retrospective cohort study found that ARBs were more effective than ACE inhibitors in reducing the risk of cognitive impairment and dementia in hypertensive patients [100]. A meta‐analysis illustrated that ARBs were more effective than ACE inhibitors in reducing the risk of cognitive impairment and dementia [101]. Thus, activating the inflammatory axis of RAAS in cardiac diseases is implicated in the development of cognitive impairment and cardiogenic dementia.

### 2.6 Sympathetic Overactivation

Sympathetic activation in heart failure is regarded as a beneficial compensatory mechanism to improve cardiac contractility and cardiac output to preserve peripheral perfusion. However, progression of heart failure and lethality [102]. Cardiac nerve growth factor, which antagonise, the sympathetic effect on the heart, is highly reduced following myocardial infarction, leading to the augmentation of heart sensitivity to the effect of sympathetic drive [102]. Therefore, reduction of sympathetic may be effective in protecting the failing heart. In 1979, Swedberg et al. [103] highlighted that β‐blocker drugs could be beneficial in the management of heart failure due to congestive cardiomyopathy by about 15 years before the first clinical trial regarding the use of carvedilol in heart failure [104]. Sympathetic overactivation in heart failure is due to activation of RAAS, which induces the central and peripheral sympathetic system [105]. In addition, sympathetic activation provokes atrial fibrillation and myocardial infarction [106]. Sympathetic overactivation in cardiac diseases leads to the dysregulation of the autonomic nervous system (ANS) by inhibiting the anti‐inflammatory parasympathetic nervous system (PNS) [102]. Moreover, prolonged sympathetic activation activates the immune cells to release pro‐inflammatory cytokines and the development of systemic inflammation and neuroinflammation [107, 108]. In turn, neuroinflammation activates the brain's cardioregulatory centre, leading to the further activation of sympathetic drive [108]. Moreover, RAAS‐induced oxidative stress promotes sympathetic activation, which also causes the development of oxidative stress [109]. Interestingly, systemic oxidative stress and inflammation‐induced BBB injury promote the transport of AngII into the brain for activation of the sympathetic centre with subsequent increasing of sympathetic drive in mice with heart failure [110]. Therefore, sympathetic overactivation in cardiac diseases through induction of oxidative stress and inflammation may induce BBB injury, which is involved in the development and progression of dementia.

**FIGURE 9:**
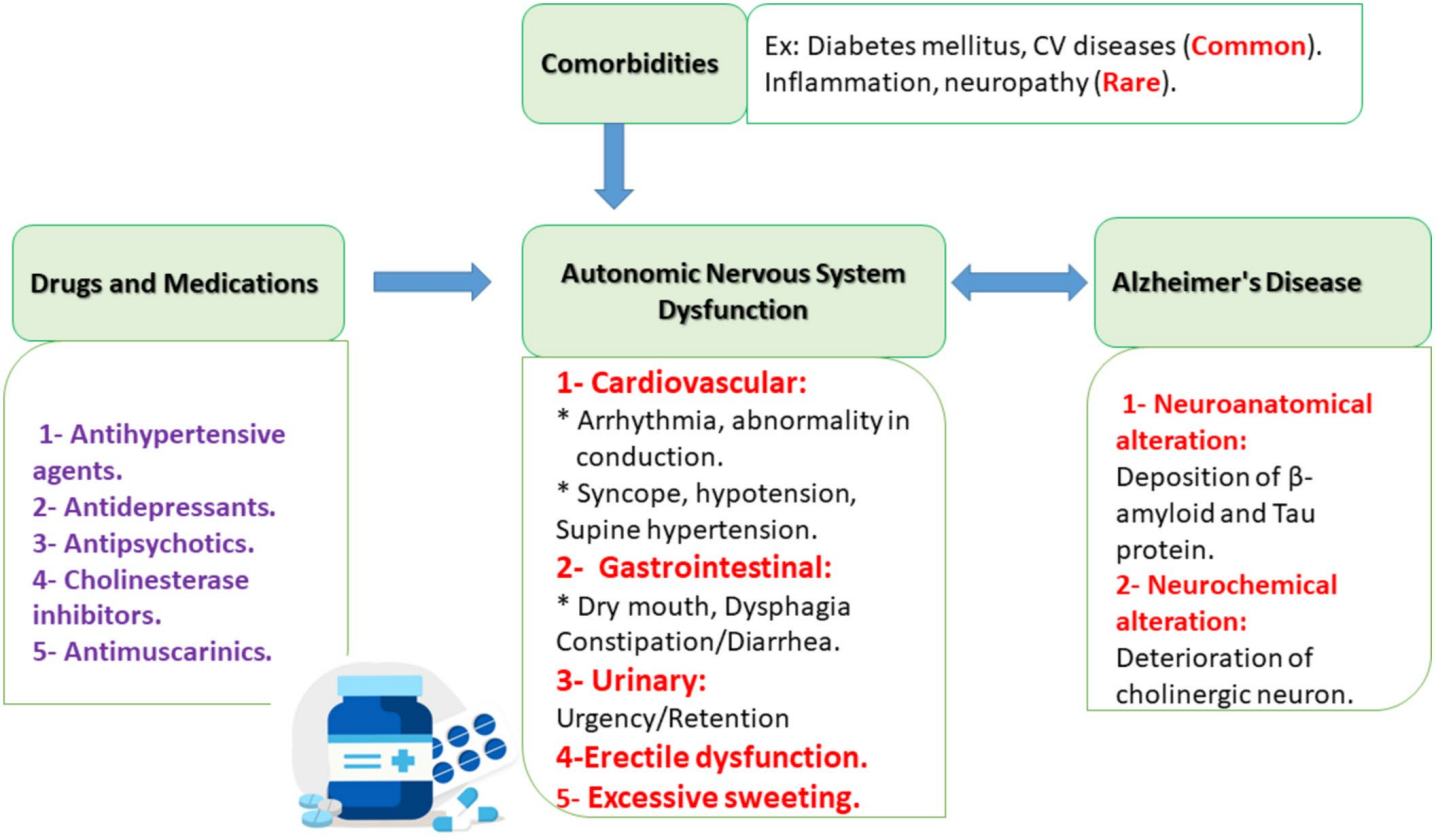
Schematic diagram showing the crosstalk between the autonomic nervous system dysfunction and the risk of dementia.

Furthermore, autonomic dysfunction is common in dementia, mainly in AD and PDD [111]. In addition, dysregulation of ANS reduced cerebral blood flow and augments chronic hypoperfusion in dementia [112]. Increasing brain sympathetic neurotransmitters promotes AD neuropathology by increasing Aβ aggregation and the formation of NFTs [113]. Inhibition of sympathetic drive by the α_2_ agonist idazoxan attenuates the progression of AD neuropathology [113]. Therefore, sympathetic overactivation in cardiac diseases is intricate in the development and progression of cardiogenic dementia (Figure 9).

Taken together, cardiogenic dementia is developing by chronic cerebral hypoperfusion‐dependent and independent mechanisms.

## 3 Conclusions

Cardiogenic dementia is a cognitive impairment associated with heart diseases such as heart failure, myocardial infarction, and atrial fibrillation. The prevalence of cognitive impairment in patients with heart failure and CAD is augmented. Moreover, myocardial infarction and subsequent systemic haemodynamic instability promote the development and progression of cardiogenic dementia. These findings designate that cardiac diseases are concerned in the development and progression of cognitive impairment.

Different mechanisms are involved in the pathogenesis of cardiogenic dementia that are chronic cerebral hypoperfusion‐dependent mechanisms or chronic cerebral hypoperfusion‐independent mechanisms. Chronic cerebral hypoperfusion is developed due to different cardiac diseases that induce the development of dementia by encouraging Aβ formation, neurodegeneration, and brain glucose hypometabolism. Systemic low‐grade inflammation in cardiac diseases may be implicated in the pathogenesis of cardiogenic dementia. Augmentation of systemic oxidative stress in different cardiac diseases is intricate in the development and progression of cardiogenic dementia. As well, BBB injury induced by cardiac diseases could be a potential mechanism in the development and progression of dementia. Furthermore, overactivated RAAS in cardiac diseases may induce the development and progression of cognitive impairment and dementia. Activation of AT_1_R is associated with BBB injury and neuroinflammation, and brain AngII contributes to the pathogenesis of vascular dementia by inducing oxidative stress, neuroinflammation, synaptic injury, and endothelial dysfunction. Both central and peripheral RAAS are associated with the progression of AD neuropathology by increasing the production and impeding the clearance of Aβ. Thus, ACE inhibitors and ARBs may reduce the pathogenesis of dementia and cognitive impairment by reducing neuronal inflammation and oxidative stress. Furthermore, autonomic dysfunction, which is common in AD and PDD, can reduce cerebral blood flow and augment chronic hypoperfusion in dementia. Increasing brain sympathetic neurotransmitters promotes AD neuropathology by increasing Aβ aggregation and the formation of NFTs. Hence, inhibition of sympathetic drive attenuates the progression of AD neuropathology. Therefore, sympathetic overactivation in cardiac diseases is intricate in the development and progression of cardiogenic dementia.

Taken together, cardiogenic dementia is developing by chronic cerebral hypoperfusion‐dependent and independent mechanisms. Additional preclinical and clinical studies are warranted in this concern.

## Author Contributions

**Nawaf AlRawili:** conceptualization (equal), validation (equal). **Ali I. Al‐Gareeb:** resources (equal), validation (equal), visualization (equal). **Maha M. Abdel‐Fattah:** writing – review and editing (equal). **Nasser A. Al‐Harchan:** writing – review and editing (equal). **Mubarak Alruwaili:** writing – original draft (equal). **Marios Papadakis:** funding acquisition (equal), writing – original draft (equal). **Athanasios Alexiou:** supervision (lead), visualization (equal), writing – original draft (equal). **Gaber El‐Saber Batiha:** writing – review and editing (equal).

## Ethics Statement

The authors have nothing to report.

## Conflicts of Interest

The authors declare no conflicts of interest.

## Data Availability

Since no new data were examined for this study, data sharing is not available to this article.
